# A comparative sequence analysis reveals a common GBD/FH3-FH1-FH2-DAD architecture in formins from *Dictyostelium*, fungi and metazoa

**DOI:** 10.1186/1471-2164-6-28

**Published:** 2005-03-01

**Authors:** Francisco Rivero, Tetsuya Muramoto, Ann-Kathrin Meyer, Hideko Urushihara, Taro QP Uyeda, Chikako Kitayama

**Affiliations:** 1 Center for Biochemistry and Center for Molecular Medicine, Medical Faculty, University of Cologne. Joseph-Stelzmann-Strasse 52, 50931 Köln, Germany; 2 Gene Function Research Center, Tsukuba Central #4, National Institute of Advanced Industrial Science and Technology (AIST), Higashi 1-1-1 Tsukuba-shi, Ibaraki 305-8562, Japan; 3 Institute of Biological Science, University of Tsukuba, Tsukuba-shi, Ibaraki 305-8572, Japan

## Abstract

**Background:**

Formins are multidomain proteins defined by a conserved FH2 (formin homology 2) domain with actin nucleation activity preceded by a proline-rich FH1 (formin homology 1) domain. Formins act as profilin-modulated processive actin nucleators conserved throughout a wide range of eukaryotes.

**Results:**

We present a detailed sequence analysis of the 10 formins (ForA to J) identified in the genome of the social amoeba *Dictyostelium discoideum*. With the exception of ForI and ForC all other formins conform to the domain structure GBD/FH3-FH1-FH2-DAD, where DAD is the Diaphanous autoinhibition domain and GBD/FH3 is the Rho GTPase-binding domain/formin homology 3 domain that we propose to represent a single domain. ForC lacks a FH1 domain, ForI lacks recognizable GBD/FH3 and DAD domains and ForA, E and J have additional unique domains. To establish the relationship between formins of *Dictyostelium *and other organisms we constructed a phylogenetic tree based on the alignment of FH2 domains. Real-time PCR was used to study the expression pattern of formin genes. Expression of *forC, D, I *and *J *increased during transition to multi-cellular stages, while the rest of genes displayed less marked developmental variations. During sexual development, expression of *forH *and *forI *displayed a significant increase in fusion competent cells.

**Conclusion:**

Our analysis allows some preliminary insight into the functionality of *Dictyostelium *formins: all isoforms might display actin nucleation activity and, with the exception of ForI, might also be susceptible to autoinhibition and to regulation by Rho GTPases. The architecture GBD/FH3-FH1-FH2-DAD appears common to almost all *Dictyostelium*, fungal and metazoan formins, for which we propose the denomination of conventional formins, and implies a common regulatory mechanism.

## Background

Eukaryotic cells rely on de novo nucleation mechanisms to generate actin filaments in order to elicit spatial and temporal remodeling of their actin cytoskeleton. Besides the Arp2/3 complex, nucleation activity has been recently demonstrated also for formins (reviewed in [1]). Formins are multidomain proteins conserved from plants to fungi and vertebrates. Their name originates from the mouse *limb deformity *gene. Mice with mutant alleles fail to form proper limbs and kidneys [2]. Subsequently, homologues were identified in Drosophila (*Diaphanous*) [3] and yeast (Bni1p and Cdc12p) [4,5]. Due to their pivotal role in the organization of the actin cytoskeleton formins are involved in processes as diverse as formation of filopodia, microspikes and lamellipodia, establishment and maintenance of cell polarity, vesicular trafficking, formation of adherens junctions, cytokinesis, embryonic development and signaling to the nucleus (reviewed in [6]).

The FH2 (formin homology 2) domain is the defining feature of all formins. It is very well conserved and is almost invariably preceded by a proline-rich region, the FH1 (formin homology 1) domain [6,7]. In vitro, the FH2 domain competes with barbed-end capping proteins and is necessary and sufficient to nucleate actin polymerization, but the FH1 domain, which interacts with profilin-actin, funnels actin to the nucleation vicinity and confers full activity to the molecule [1]. Contrary to the Arp2/3 complex, which nucleates a new filament on the side of a preexisting filament, remains attached to the pointed end of the new filament and generates branched networks [8], the FH2 domain binds and stays associated to the barbed end, giving rise to unbranched filaments [9-11]. The crystal structure of the FH2 domain of two formins, Bni1p and mDia1, has been recently solved. Its fold is almost entirely α-helical and forms a ring-shaped flexible but stable dimer that caps the barbed end and allows processive elongation of the actin filament [12,13]. The FH1 domain is also a binding site for diverse SH3-domain containing proteins like Src-like non-receptor tyrosine kinases, WISH (WASP-interacting SH3 protein) and IRSp53 (insulin receptor substrate) in mammals, and Hof1p in yeast [6].

In most fungal and metazoan formins the FH1-FH2 core is accompanied by a less well conserved N-terminal FH3 (formin homology 3) domain involved in targeting [14]. In plants targeting might be mediated by membrane insertion signals or PTEN (phosphatase and tensin)-related domains [15,16]. Some formins, the so called Diaphanous-related formins, are able to interact with activated Rho GTPases through a poorly defined N-terminal Rho GTPase binding domain (GBD) that overlaps with the FH3 domain [6,7]. This binding releases the intramolecular inhibitory interaction between the GBD and a C-terminal Diaphanous autoregulatory domain (DAD) and renders the protein active [10,17].

The social amoeba *Dictyostelium discoideum *is an attractive model organism to investigate the components of the actin cytoskeleton and the signaling pathways involved in its regulation [18,19]. *Dictyostelium *amoebae are equipped with a complex actin cytoskeleton that endows the cells with motile behavior comparable to that of human leukocytes. In fact, a genome-wide survey revealed that the repertoire of cytoskeletal components of *Dictyostelium *is more similar to metazoa followed by fungi than to plants (Eichinger, et al., submitted). In *Dictyostelium*, nine formins have been previously identified but only three of them have been characterized to some extent [20]. Mutants lacking ForA, ForB or both showed no detectable phenotype, whereas disruption of the gene encoding ForC, which is expressed predominantly at late developmental stages, led to a cell autonomous developmental defect with the formation of aberrant fruiting bodies, suggesting this formin mediates actin remodeling during multicellular stages. *In vivo *experiments with GFP fusions showed that the N-terminal region of ForC targets the protein to places of active actin reorganization, like macropinosomes, phagocytic cups and cell-to-cell contacts [20].

We have made use of the information released by the *Dictyostelium *sequencing projects in order to achieve a complete inventory of formin genes. A detailed sequence analysis of the 10 formins identified revealed that, with the exception of ForI and ForC, all other formins conform to the domain structure GBD/FH3-FH1-FH2-DAD present in almost all fungal and metazoan formins, for which we propose the denomination of conventional formins. Our sequence analysis also indicates that the GBD and FH3 domains constitute a single domain also found in two *Dictyostelium *RasGEFs (guanine nucleotide exchange factors). The expression pattern of formin genes during asexual and sexual development was studied using real-time PCR. Our analysis allows some preliminary insight into the functionality of *Dictyostelium *formins: all isoforms might display actin nucleation activity and, with the exception of ForI, might also be susceptible to autoinhibition and regulation by Rho GTPases.

## Results

### Sequence analysis of *Dictyostelium *formin genes

In a previous publication 9 genes that potentially encode proteins of the formin family were identified in *Dictyostelium *[20]. For some of the formins (ForA through D and ForF) full length sequences were available, whereas for the rest N- and C-terminal sequences were missing. For a complete analysis of this family in *Dictyostelium *we sought to exploit the available databases in order to achieve a complete inventory of formin genes in its entire length. The sequences already reported by Kitayama et al. [20] were used as queries for Blast searches of the *Dictyostelium *genomic DNA database. This allowed assembly of complete genomic sequence for *forA *through *forI*. In order to verify the predicted amino acid sequence for each formin, Blast searches were performed against the *Dictyostelium *EST database. In cases where no EST sequences were available, like *forG *and *forI*, introns were verified after RT-PCR.

Inspection of the EST sequences led to the identification of one more formin gene, *forJ*, whose genomic sequence was also retrieved and inspected. Recent completion of the assembly of the *Dictyostelium *genome allowed us to confirm our gene predictions and map each formin gene to its corresponding chromosome locus (Eichinger et al., submitted). Formin genes are dispersed all over the six chromosomes (each chromosome harbors at least one formin gene), and in no case two or more genes are placed adjacent to each other (Table 1).

**Table 1:** Features of *Dictyostelium *discoideum formins Sequences can be accessed through the Dictybase identifier at

**Gene**	**Dictybase ID**	**Chromosome**	**Number of introns**	**Numer of residues**
*forA*	DDB0214996	3	5	1218
*forB*	DDB0215000	3	1	1126
*forC*	DDB0191362	5	2*	1158
*forD*	DDB0205290	3	3	1214
*forE*	DDB0190413	1	0	1561
*forF*	DDB0188569	5	1	1220
*forG*	DDB0169087	2	1	1074
*forH*	DDB0186588	4	3	1087
*forI*	DDB0186053	4	2	935
*forJ*	DDB0183855	6	1	2546

* One intron upstream of the start codon.

**Figure 1:**
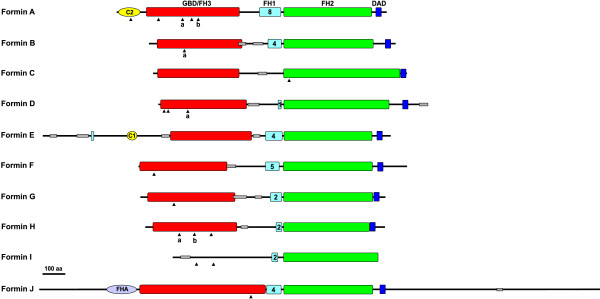
**Domain organization of *Dictyostelium *formins. **With few exceptions, *Dictyostelium *formins conform to the domain structure GBD/FH3-FH1-FH2-DAD. Diagrams have been aligned with the FH2 domain. Regions with high probability of coiled coil structure are depicted as thin gray rectangles. C1 and C2 correspond to protein kinase C conserved regions 1 and 2, respectively. FHA is a forkhead-associated domain. Numbers inside the FH1 boxes indicate the number of XPPPPP motifs. Triangles denote the position of introns. Only introns placed in coding regions are shown. Intron positions shared by two or more genes have been labeled with letters.

With the exception of *forE*, all other formin genes are interrupted by one or more introns, which are generally placed in the 5' half of the sequence, upstream of the region encoding the FH1 domain (Fig. 1, arrowheads). Only in *forC *is an intron placed in the region encoding the FH2 domain. *ForC *is also the only case where an intron was identified upstream of the start codon. *Dictyostelium *formin genes do not appear to undergo alternative splicing, at least within the coding region. This is in contrast to metazoan and plant formins, where alternative splicing gives rise to a large number of variants that frequently differ in their pattern of tissue distribution and interaction with binding partners.

**Figure 4:**
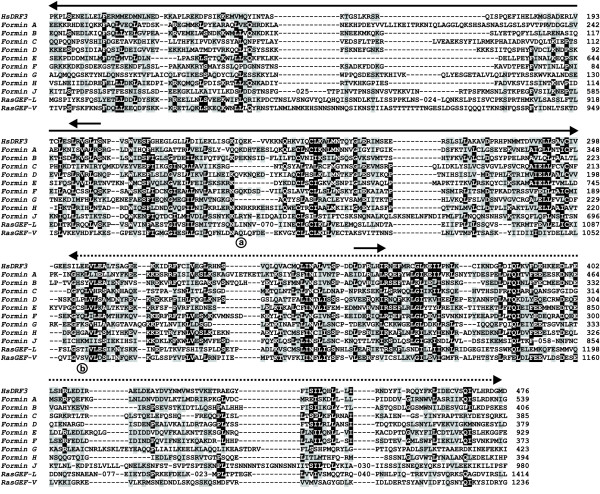
**Multiple alignment of the GBD/FH3 domains of *Dictyostelium *formins and two RasGEFs **Amino acid sequences were aligned with ClustalX and the output file was subsequently edited manually. In addition to nine *Dictyostelium *formins, a GBD/FH3 domain was identified also at the N-terminus of RasGEF-L and RasGEF-V. The sequence of the human DRF3 has been included for reference. Dashes indicate gaps introduced for optimal alignment. In some places extensive repetitive stretches have been removed and replaced by a figure indicating the number of residues omitted. Residues identical or similar in at least 40% of the sequences are boxed in black or gray, respectively. Continuous and discontinuous lines indicate, respectively, the extension of the GBD and FH3 domains as defined in the Pfam database. Short arrows indicate boundaries of the FH3 domain as proposed by Petersen et al. [14]. Conserved intron positions are labeled a and b (see Fig. 1).

Only two intron positions are conserved among *Dictyostelium *formin genes (Figs. 1 and 4). Intron a is conserved in *forA*, *forB*, *forD *and *forH*, whereas intron b is conserved in *forB *and *forH*. The conserved FH3-FH1-FH2 core domain composition (see below) along with these two intron positions underscore the view that all *Dictyostelium *formin genes might have arisen from a common ancestor gene. After duplications and divergence from this ancestral formin gene introns were acquired or lost and additional domains and extensions were appended to some genes.

### Domain structure of *Dictyostelium *formins: the FH2 domain

The domain structure and topology of all ten *Dictyostelium *formins was determined by means of bioinformatics tools and visual inspection. Although formins vary considerably in length (935 residues of ForI versus 2546 of ForJ), with few exceptions they have in common a core of about 1100 residues that harbors a GBD/FH3-FH1-FH2-DAD structure characteristic of most fungal and metazoan formins (Fig. 1). To better appreciate the relationships among the members of the *Dictyostelium *formin family and to analyze the requirements for their function, we have generated multiple alignments of the FH2-DAD domains as well as the GBD/FH3 domain.

**Figure 2:**
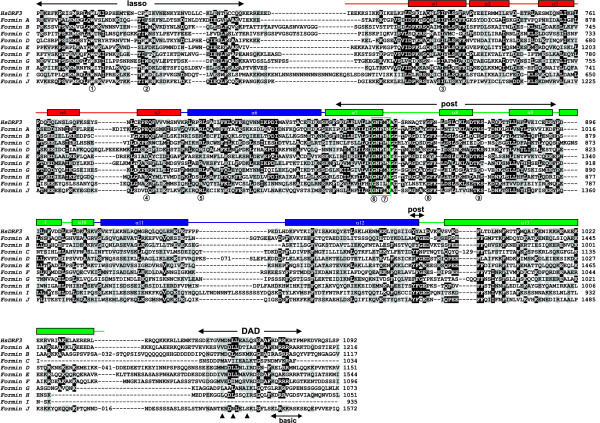
**Multiple alignment of FH2 and DAD domains of *Dictyostelium *formins. **Amino acid sequences were aligned with ClustalX and the output file was subsequently edited manually. The sequence of the human Diaphanous-related formin 3 has been included for reference. Dashes indicate gaps introduced for optimal alignment. In some places extensive repetitive stretches have been removed and replaced by a figure indicating the number of residues omitted. Residues identical or similar in at least 40% of the sequences are boxed in black or gray, respectively. Secondary structure elements as determined for mouse Dia1 core FH2 domain [12] are indicated on top of the aligned sequences. Color coding denotes the N-terminal knob subdomain (red), three-helix-bundle (blue) and FH2 motif post-containg region (green). Regions involved in the formation of the lasso/post dimer interface, as determined for Bni1p [13] are also indicated, as well as the highly conserved GNY/FMN motif (boxed). Conserved residues discussed in the text are indicated by circles and are numbered consecutively. Below the DAD region triangles indicate conserved residues discussed in the text.

The FH2 domain is the best conserved domain of formins (Fig. 2). In general, the FH2 domain is about 400 residues long. Some *Dictyostelium *formins (ForC, D and I) have one or more stretches of intervening repetitive sequences of variable length rich in Arg, Gln or Ser. Such repetitive sequences are characteristic of many *Dictyostelium *genes. The crystal structure of the FH2 domain of two formins, Bni1p and mDia1, has been recently solved [12,13]. We will consider the FH2 domains of *Dictyostelium *formins in the context of these two structures. The FH2 domain fold is almost entirely α-helical. It is a stable dimer that forms a closed parallelogram-shaped ring. The structure of this domain can be subdivided into subdomains. At the N-terminus a so-called lasso is connected to a globular knob (helices α1 to α5 in red in Fig. 2) by a linker of variable length. The knob is followed by a three helix bundle with a coiled-coil structure (α6, α11 and α12 in blue). The C-terminal subdomain (helices α7 to α10 and α13 in green) forms a so-called post. The lasso subdomain of one subunit encircles the post subdomain of the other subunit in a dimer. The post also harbors the GNY/FMN sequence motif that originally defined the FH2 domain (box at the end of helix α7) [21]. Residues of the lasso/post interface are highly conserved, particularly Trp1 and 2 (substituted by Phe in ForB, E and F) that insert into hydrophobic pockets in the post flanked by Gly residues 6 and 8 (Fig. 2).

All residues of the sequence motif GNY/FMN participate in dimerization. This motif is also highly conserved in almost all *Dictyostelium *formins (NY is substituted by SI in ForI) but the important methionine residue (Met7) [12] is present only in ForG and ForJ and is substituted by other hydrophobic residues in the rest of the *Dictyostelium *formins as well as in members of the FHOD (formin homology domain containing protein) and plant class1 subfamilies.

Also very conserved are some residues probably involved in binding to actin, like Ile3 (absolutely conserved) in the N-terminal subdomain and Lys9 in the post region (substituted by Arg in ForB and ForH). Mutation of these residues in Bni1p to Ala and Asp, respectively, abolished actin nucleation and barbed end capping activity of the FH2 domain [13], and replacement of Lys9 and two adjacent Lys residues by Ala abolished alignment of microtubules and bundling of F-actin induced by activated mDia1 [22]. Other conserved residues are Asp4 (or the conservative substitution by Glu in most of the *Dictyostelium *formins) and Arg5 (substituted by Lys in ForB and ForE). These residues were found mutated in temperature-sensitive yeast mutants [23,24], and they probably participate in stabilization of the knob region [13].

In summary, all essential residues in the FH2 domain revealed by structural and functional studies in metazoan and fungal formins are conserved in *Dictyostelium *formins, indicating that all ten formins might be functional actin nucleators.

### *Dictyostelium *formins in the context of other organisms

In order to establish the relationship between formins of *Dictyostelium *and other organisms and to investigate whether different species share subfamilies of formins, we constructed a phylogenetic tree based on the alignment of complete sets of sequences of FH2 domains from selected organisms, including representatives of fungi, plants, invertebrates and vertebrates. We retrieved sequences of already characterized formins and additionally we made a search of further available sequences through the SMART server with the FH2 domain as query. Appart from the ten sequences of *Dictyostelium*, we collected a total of 62 sequences, 21 from plants, 5 from yeasts, 6 from *D. melanogaster*, 6 from *C. elegans *and 14 from human. Taking into account that some genes might not have been predicted accurately and that predicted proteins not supported by EST sequences were not considered for our analysis, further metazoan formins, especially from human, most probably went unidentified in our search.

**Figure 3:**
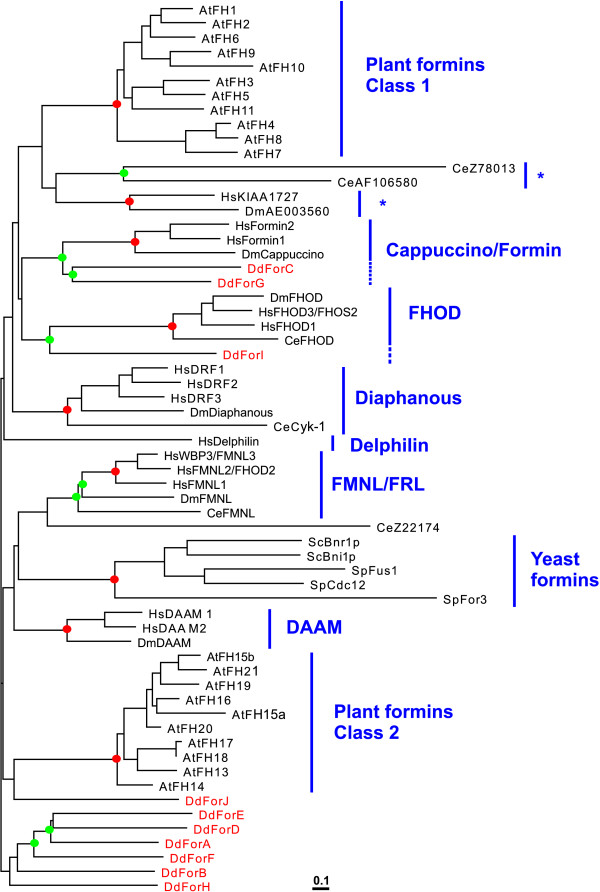
**Phylogenetic tree of FH2 domains of formins from *Dictyostelium *and other organisms. **Amino acid sequences of the FH2 domains (core and lasso region) were aligned with ClustalX and the output file was subsequently edited manually. A bootstrapped unrooted phylogenetic tree was constructed as described in the Methods section. *Dictyostelium *members are indicated in red. The other organisms considered are *Arabidopsis thaliana *(At), *Saccharomyces cerevisiae *(Sc), *Schizosaccharomyces pombe *(Sp), *Drosophila melanogaster *(Dm), *Caenorhabditis elegans *(Ce) and *Homo sapiens *(Hs). Nodes supported by either >75% or >50% bootstraps have been marked with red or green circles, respectively. For simplicity, nodes outside of a cluster supported by >50% bootstraps have not been indicated. Asterisks denote novel formin subfamilies. The scale bar indicates percent substitutions.

The phylogenetic tree (Fig. 3) supports the high degree of conservation of the FH2 domain, as becomes evident from the homogeneous branch length for most of the sequences. Yeast formins and some *C. elegans *members are more divergent. The phylogenetic analysis reveals clustering of most formins into well defined classes. Yeast formins form a separate class whereas plant formins significantly group into any of two classes. Metazoan formins do not constitute a single cluster, rather they distribute into a number of subfamilies. The FHOD, Diaphanous and FMNL (formin in leukocytes) subfamilies have representatives in human, *D. melanogaster *and *C. elegans*. The Cappuccino/Formin and DAAM (Dishevelled-asssociated activator of morphogenesis) subfamilies, as well as a novel subfamily, is present in human and *D. melanogaster*, but seems to be absent in *C. elegans*. Delphilin constitutes a subfamily with a unique member present only in human. Finally, *C. elegans *has some additional divergent formins apparently unique to this organism.

On average, *Dictyostelium *formins are 45.5% similar (23.8% identical) to each other, with ForC being only slightly more divergent (40.0%/20.4% similarity/identity to the rest of *Dictyostelium *formins). A comparable degree of similarity (identity) was found to members of several subfamilies of metazoan formins, and ranged between 40% (20%) and 48% (24%). Similarity (identity) to plant and yeast formins was lower: 38% (19%) and 36% (17%) respectively. *Dictyostelium *ForC and ForG cluster together with the Cappuccino/Formin group (75% bootstraps), whereas ForI very weakly clusters with the FHOD subfamily (53% bootstraps). However, taking into account that the FH2 domain is highly conserved, the position of these three *Dictyostelium *formins in the tree does not necessarily mean functional relationship with the mammalian counterpart, because other domains are probably responsible for diversity of localization and function. Bootstrapping does not support a significant clustering of the rest of the *Dictyostelium *formins, and only few members cluster together with a reasonably high number of bootstraps (ForE, D, A and F, 51% bootstraps).

### Domain structure of *Dictyostelium *formins: FH1, FH3 and other domains

The FH1 domain is a proline-rich region situated immediately upstream of the FH2 domain. It is present in almost all known formins, including that of *Dictyostelium*, with the notable exception of ForC. The length of the FH1 domains is very variable among formins (10 to >500 amino acids). It constitutes a binding site for the actin monomer binding protein profilin, as well as for SH3 and WW domain containing signaling proteins [25,26]. Binding to profilin is well established for a large number of formin proteins and might take place through type 1 proline-rich motifs with the sequence XPPPPP, where X is usually Gly, Leu, Ile or Ser. *Dictyostelium *formis have a variable number of these motifs, between 1 in ForD and 8 in ForA (Fig. 1). In most cases Gly occupies the X position. In general the motifs are separated by a short stretch of up to five residues, two or more of them usually glycines. In some formins, like ForA and ForF, the proline-rich motifs might be the product of internal duplications. ForE has one additional short proline-rich region located at the N-terminus of the protein.

The FH3 domain was initially identified and characterized in the yeast formin Fus1p as a region consisting of three blocks of similarity in the same relative order in several formins [14]. It is less well conserved than the FH2 domain and is thought to be important for determining the intracellular localization of formins. Two domains of the Pfam database are recognized in this region that overlap with the FH3 domain of Petersen and co-workers [14], the Diaphanous GTPase-binding domain (PF06371) and the Diaphanous FH3 domain (PF06367). Automatic domain analysis identified a GBD and a FH3 domain in ForA, B, D, E, F and H. In ForC and ForJ a GBD was identified with confidence values slightly below the default threshold of the SMART tool. This was also the case for a FH3 domain in ForG and ForJ. A multiple alignment of the N-terminus of *Dictyostelium *formins with metazoan and fungal homologues revealed a homology region of approximately 380 residues in all *Dictyostelium *formins with the exception of ForI (Figs. 1 and 4). We will consider this region as a single GBD/FH3 domain (see discussion). In ForJ this domain is considerably longer due to stretches of intervening repetitive sequences rich in Arg and Ser residues. On average the GBD/FH3 domain of *Dictyostelium *formins displays 39% similarity to that of human DRF3 taken as reference for figure 4. Interestingly, inspection of the *Dictyostelium *genome for proteins with a GBD as defined by Pfam PF06371 yielded two genes encoding RasGEF proteins of identical domain composition, RasGEF-L and RasGEF-V. Both proteins harbor a complete GBD/FH3 domain that is 35% similar to that of human DRF3 and constitute the first case where this domain is observed outside of a formin.

**Figure 5:**
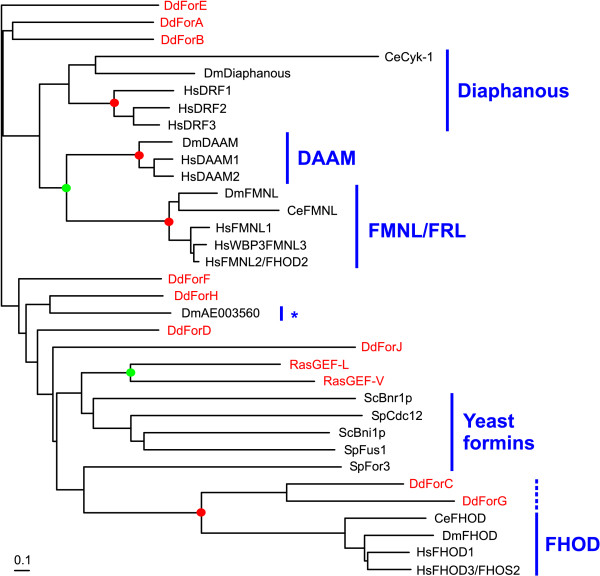
**Phylogenetic tree of the GBD/FH3 domains of formins and two RasGEFs from *Dictyostelium *and formins from other organisms. **Amino acid sequences of the GBD/FH3 domains were aligned with ClustalX and the output file was subsequently edited manually and intervening sequences between blocks of high similarity were removed. The sequence available for DmAE003560 only contains a FH3 domain and a short part of the GBD. A bootstrapped unrooted phylogenetic tree was constructed as described in the Methods section. *Dictyostelium *members are indicated in red. The other organisms considered are as in the legend to figure 3. Labeling is also as in the legend to figure 3.

We constructed a phylogenetic tree based on a multiple alignment of the GBD/FH3 domain of *Dictyostelium *formins (except ForI), RasGEFs, fungal formins and members of the Diaphanous, DAAM, FMNL and FHOD subfamilies (Fig. 5). With few exceptions automatic domain analysis identified GBD and FH3 domains in the metazoan and fungal formins. For example, a weak GBD was identified in *D. melanogaster *and *C. elegans *FHOD, but not in the human homologs, and conversely, a weak FH3 domain was identified in HsFHOD3 but not in other members of the subfamily. In those cases the missing domain could be reliably identified in multiple alignments. We could not identify a GBD/FH3 domain in members of the cappucino/formin subfamily. DmAE003560 has a FH3 domain and a short piece of a GBD but, interestingly, the human homolog KIAA1727 completely lacks an N-terminal region and starts at the FH1 domain. Inspection of the sequence databases did not allow clearing whether the available sequences correspond to spliced variants of longer proteins. The multiple alignment of the GBD/FH3 domain showed several blocks where similarity is higher among sequences, generally in the central part of the domain (Fig. 4). In many cases these blocks are separated by intervening stretches of variable length in the different subfamilies. We removed these insertions from our alignment prior to calculating the tree. The phylogenetic tree showed significant clustering of members of the respective metazoan subfamilies, and additionally the FMNL and DAAM subfamilies clustered together (73% bootstraps). Bootstrap analysis did not support clustering of fungal or *Dictyostelium *sequences into distinct classes, but interestingly, ForC and ForG significantly clustered with the FHOD family (92% bootstraps).

The DAD immediately follows the FH2 domain and is required for autoinhibition by intramolecular interaction with the N-terminus of formins [21]. Inspection of the multiple alignment of the C-terminus of *Dictyostelium *formins revealed a DAD in all members with the exception of ForI (Figs. 1 and 2). This formin ends abruptly at the last α-helix of the FH2 domain. In all cases the DAD was placed in the vicinity of and no more than approximately 60 residues beyond the FH2 domain. The DAD is composed of two sections, a core leucine-rich sequence and a short stretch of basic residues. Both elements are present in the DAD of most *Dictyostelium *formins, in particular three hydrophobic residues shown to be required for activity in mouse Dia2 (indicated by triangles in Fig. 2) [17]. In ForJ, where these residues are substituted by polar or charged aminoacids, the DAD might not be functional.

Like metazoan and fungal formins, most *Dictyostelium *formins have predicted coiled-coil regions adjacent to the FH3 domain that could act as protein-protein interfaces for yet unidentified ligands (Fig. 1). For example, in mammalian formin1 this region constitutes the binding site of α-catenin and is involved in recruitment of formin1 to nascent adherens junctions [27] and in Bni1p the coiled coil region harbors the binding site for Spa2, a protein involved in recruitment of Bni1p to the bud cortex [28]. A few *Dictyostelium *formins have additional predicted coiled coil regions upstream of the FH3 domain (ForE and ForI) or downstream of the FH2 domain (ForD and ForJ) that might constitute potential protein interaction sites with regulatory or targeting functions.

Three *Dictyostelium *formins have additional recognizable domains at their N-terminus (Fig. 1). ForA has a C2 (protein kinase C conserved region 2) domain. This domain, present in phospholipases, protein kinases C, synaptotagmins and diverse other proteins, is thought to be involved in calcium-dependent phospholipid binding [29]. ForE has a C1 (protein kinase C conserved region 1) domain, a cysteine-rich region involved in zinc-dependent binding to diacylglycerol [30]. Finally, ForJ has a FHA (forkhead-associated) domain, a phospho-specific protein-protein interaction motif found in nuclear proteins [31]. None of these domains are found in formins from other organisms. ForJ is the only *Dictyostelium *formin with a long C-terminal extension. Similar extensions, although of unrelated sequence, can be observed in formins from other organisms, like yeast Cdc12 and For3, *C. elegans *Cyk-1 and AF106580, *D. melanogaster *AE003560 and human KIAA1727.

### Expression analysis

**Figure 6:**
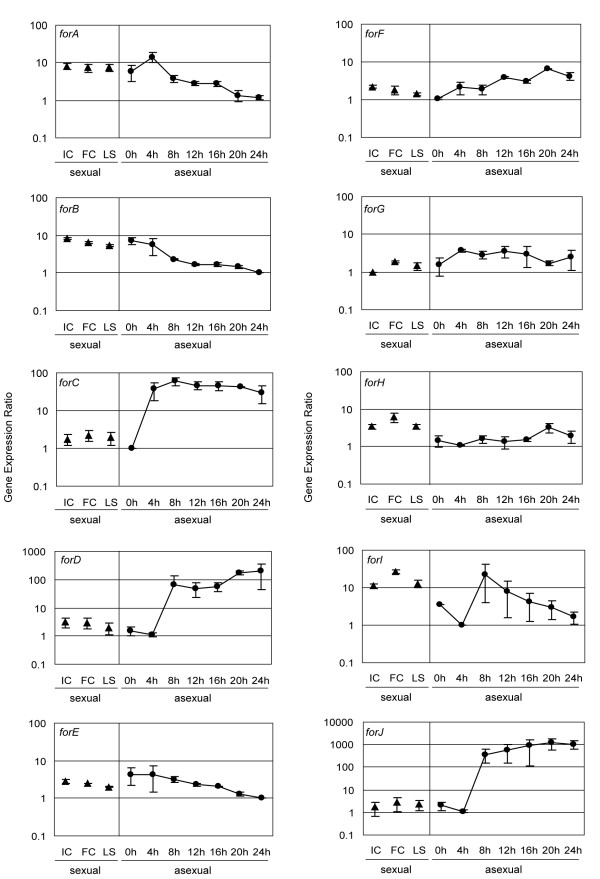
**Expression analysis of *Dictyostelium *formin genes. **Expression analysis was performed using quantitative real time PCR on two independently isolated mRNA samples both in sexual and asexual developmental stages. Average and standard deviation of two independent expression ratios obtained from independent cDNA samples are shown. IC, fusion incompetent cells; FC, fusion competent cells; LS, light submerged cells.

*Dictyostelium *cells can propagate following either an asexual or a sexual life cycle. Characteristic of the asexual life cycle is the transition from single cell amoebae to a multicellular fruiting body consisting of at least two differentiated cell types. In the sexual life cycle some amoebae become sexually mature under dark and submerged conditions, fuse and form macrocysts. Either life cycle involves coordinated transcription of certain sets of genes. We have used quantitative real-time PCR to study the expression of the formin genes during sexual and asexual development (Fig. 6).

The expression patterns observed during asexual development can be classified into two major groups. Expression of *forC, D, I *and *J *displayed an increase during transition to multi-cellular stages, and except for *forI*, levels remained constantly high throughout the rest of development. The rest of genes displayed less marked developmental variations, and expression was either kept at constant levels (*forG *and *H*) or gradually increased (*forF*) or decreased (*forA, B *and *E*) after the onset of development.

When expression was analyzed during sexual development only *forH *and *forI *displayed a significant increase of about 3-fold in fusion competent cells compared to fusion incompetent cells. Cells cultured in light submerged conditions have a reduced sexual fusion competency [32,33]. In parallel with this, *forH *and *forI *were enriched in fusion competent compared to light submerged cells, indicating that this enrichment is related to the acquisition of the fusion competence rather than to the submerged condition that was included to induce the fusion competence.

## Discussion

**Figure 7:**
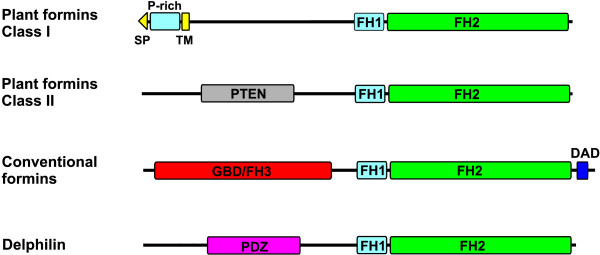
**Classification of formins according to structural and functional elements. **Most formins of metazoans as well as formins of *Dictyostelium *and fungi can be classified as conventional formins, with a GBD/FH3-FH1-FH2-DAD structure, although in particular members or alternatively spliced variants a domain (but never the FH2) might be absent. Plant formins can be grouped into one of two classes. Delphilin is an unconventional formin lacking GBD/FH3 and DAD only found in metazoa. Formins are not drawn to scale. SP, signal peptide. TM, transmembrane region.

We have performed a detailed sequence and expression analysis of the formin family of *Dictyostelium*, which in this organism comprises 10 genes. A comparison of the domain composition of formins from diverse phyla allows their grouping into four major classes (Fig. 7). In general, *Dictyostelium *formins can be grouped within the class of what we designate conventional formins (see below), which includes all fungal and almost all metazoan formins. This is in agreement with a genome wide analysis that places *Dictyostelium *closer to fungi and metazoa than to plants (Eichinger et al., submitted).

With very few exceptions all formins have in common a FH2 domain immediately preceded by a FH1 domain. The FH1-FH2 combination constitutes the minimal core that is fully functional in terms of actin nucleation and elongation activity (reviewed in [1]). This FH1-FH2 core is very ancient, and its remarkable degree of conservation points at an essential role within the cell. The diverse formin classes differ in their N-terminal regions, which have regulatory and targeting roles. Plant formins characteristically lack GBD/FH3 and DAD domains and there is no evidence for an interaction with Rop GTPases. In plants the N-terminus is unrelated to that of other organisms. Class 1 plant formins are integral membrane proteins by virtue of a signal peptide or membrane anchor followed by a transmembrane domain, whereas some class 2 plant formins have a PTEN-related domain [15,16]. Conventional formins have characteristically a GBD/FH3 domain at the N-terminus. Together with the DAD region at the very C-terminus this domain confers in most cases regulatable autoinhibition through binding of activated Rho GTPases. Finally, Delphilin is a variation only present in vertebrates. Instead of a GBD/FH3 domain it has a PDZ domain that interacts with a glutamate receptor, and it has been proposed that receptor binding causes activation of this formin [6,34].

### The GBD/FH3 domain: a targeting and regulation domain

Our sequence analysis defines a putative GBD/FH3 domain in most *Dictyostelium *as well as fungal and metazoan formins. The two domains identified in the Pfam database at the N-terminus of several formins, the Diaphanous GTPase-binding domain and the Diaphanous FH3 domain, overlap with the FH3 domain proposed initially by Petersen et al. [14]. This distinction is apparently based on reports on binding of activated Rho GTPases, however the boundaries of each domain have not been defined experimentally. We propose that these two regions constitute a single domain for two reasons. First, when present, these two domains as defined in the Pfam database invariably appear adjacent to each other and are separated by only very few residues. Some cases of sequences where only a FH3 domain is present correspond to alternatively spliced variants of proteins that in their full length possess GBD and FH3 domains. This is the case for example of HsDRF3 [35]. Second, the GBD/FH3 domain appears as a single block in two formin-unrelated proteins of *Dictyostelium*, indicating that the domain was shuffled as a unit during remodeling of the genome (Fig. 4).

The role of the GBD/FH3 domain appears to be twofold. On one hand the N-terminal region of formins is involved in subcellular localization through interaction with diverse targets. For instance, the N-terminus of yeast Fus-1 is responsible for recruitment to the projection tip during conjugation [14]. In mouse Dia3 the analogous region is required for localization at mitotic spindles [36]. In *Dictyostelium *an N-terminal fragment that encompasses most of the GBD/FH3 domain of ForC is sufficient for targeting to crowns and macropinosomes [20]. The N-terminus appears thus as a major determinant of localization and therefore function of formins. The low degree of sequence conservation of this region might correlate with the diversity of binding partners, not only Rho GTPases, and subcellular localization patterns described. On the other hand the GBD/FH3 domain is involved in regulation of activation by releasing of an intramolecular interaction between the DAD and the N-terminus, as initially proposed by Watanabe et al. [21]. As already mentioned, the boundaries of the GBD region remain poorly defined and while a CRIB-like (Cdc and Rac interactive binding) region has been described in mammalian DRF [37], such motif cannot be identified in any other formin, whether regulated by Rho GTPases or not.

Although initially not appreciated [6], with very few exceptions a GBD/FH3, a DAD or both can be identified in almost all conventional formins, including all fungal formins, FMNL, FHOD and DAAM ([11,14,38-40] and Figs. 4 and 5). Although a FH3 domain was reported also in cappuccino and in one alternatively splice variant of mouse formin 1 (formin1 IV) [14], we were not able to identify a GBD/FH3 domain in these proteins. We cannot exclude that a strongly divergent GBD/FH3 be present in members of this subfamily. In fact, cappuccino interacts with activated RhoA [41], and the N and C-terminal segments of formin1 (IV) interact with each other [27], two features characteristic of conventinal formins.

The designation Diaphanous-related formin has been applied to those formins that interact with activated Rho GTPases [7]. However, the number of formins shown to posses this property is increasing, and includes to date at least one member of each family of conventional formins as well as *Dictyostelium *formins (our unpublished data). We therefore propose the use of the name conventional formins for those subfamilies with the general structure GBD/FH3-FH1-FH2, although in particular members or in alternatively spliced variants a domain (but never the FH2) might be absent, indicating whether the protein is Rho-regulated where documented experimentally. The name Diaphanous-related formin should be restricted to the metazoan members of the Diaphanous subfamily, like human DRF1 to 3.

### Functionality and roles of *Dictyostelium *formins

Functional data on *Dictyostelium *formins is scarce. Only three isoforms, formins A, B and C have been characterized to some extent. Mutants lacking ForA, ForB or both showed no detectable phenotype, whereas deletion of *forC *led to formation of aberrant fruiting bodies with short stalks and unlifted sori, suggesting this formin mediates actin remodeling during multicellular stages [20]. *Dictyostelium *formins are expected to be functional according to their highly conserved FH1-FH2 structure; therefore a certain degree of functional redundancy is expected. However, diversity might arise through specific targeting and activation by Rho GTPases conferred by the GBD/FH3 domain, through interaction of specific SH3-domain containing proteins with the FH1 domain and by virtue of unique additional domains. These issues need to be addressed experimentally in the future.

ForC and ForI might be exceptions in terms of regulation. ForC lacks a FH1 domain and consequently does not bind to profilins [20]. Although the FH2 domain is necessary and sufficient for nucleation, FH2-induced nucleation is very slow and requires binding of profilin to the FH1 domain for full functionality [9-11]. While other scenarios are possible, in the case of ForC fueling of the actin polymerization process by profilin-actin might be furnished by heterodimerization with another formin possesing an FH1 domain. Regarding ForI, that lacks GBD/FH3 and DAD domains, it is not clear how this isoform could be regulated.

Three *Dictyostelium *formins have domains at their N-termini that are not found in other formins and might confer unique additional functions or ways of regulation or targeting. The C2 and C1 domains of ForA and ForE, respectively, might regulate activation or targeting of the molecule through interaction with specific lipids [29,30], while the FHA domain of ForJ might be involved in interactions with components of the cell nucleus [31]. In general, well defined domains others than the ones characteristic of formins are very rare. Most plant class 1 formins carry transmembrane domains and proline-rich regions in their N-termini that together might mediate anchorage of actin nucleation sites to the cell wall across the plasma membrane [15,16] and the PTEN-related domain of some class 2 plant formins might also be involved in membrane anchoring [16]. Apart from Delphilin (see above) we have identified only one more case of additional domains in metazoan formins, CeZ22171. This protein, that also lacks a FH1 domain, has a zinc finger domain and might be involved in nucleic acid interactions. The C-terminal extensions found in ForJ and several other fungal and metazoan formins also probably harbor recognition sites for additional binding partners that remain to be identified.

Functional diversity might also be related to different patterns of local and temporal gene expression. Our gene expression analyses also suggest specific roles during asexual and sexual development. Four genes in particular, *forC, D, I *and *J*, displayed an increase in expression during transition to multi-cellular stages. During this phase cells acquire aggregation competence in parallel with maturation of signaling pathways involved in remodeling of the cytoskeleton. At least for *forC *gene expression data correlate with a developmental role, as mentioned above [20]. ForH and ForI might play specific roles during sexual development, based alone on their patterns of gene expression. Interestingly, expression of *rac1b *and *racF2 *was found increased during the analysis of a gamete-enriched cDNA library [33]. It is therefore conceivable that one or more formins, irrespective of their expression pattern, play roles during sexual development upon activation by those GTPases.

## Conclusion

The social amoeba *Dictyostelium discoideum *expresses 10 formins that with few exceptions conform to the domain structure GBD/FH3-FH1-FH2-DAD. This arhitecture and the high degree of conservation of the FH2 domain allow some preliminary conclusions about the functionality of *Dictyostelium *formins: all isoforms may display actin nucleation activity and, with the exception of ForI, may also be susceptible to autoinhibition and to regulation by Rho GTPases. Although functional redundancy may be expected to occur to some extent among *Dictyostelium *formins, specific roles may be conferred by the GBD/FH3 domain, which is less well conserved than the FH2 domain, and by specific patterns of gene expression during asexual and sexual development.

We propose four major classes of formins based on a comparison of the domain composition of proteins from diverse phyla. *Dictyostelium*, fungal and most metazoan formins can be grouped within the class of what we designate conventional formins, characterized by the structure GBD/FH3-FH1-FH2-DAD. The GBD and FH3 domains, whose boundaries had not been defined previously, probably constitute a single domain. The architecture shared by conventional formins implies a common regulatory mechanism based on autoinhibition through intramoleculr interaction of the GBD/FH3 and the DAD domains and activation through release of this interaction upon binding of Rho GTPases. Formins of the other classes (plant formins and Delphilin) lack GBD/FH3 and DAD domains and must therefore have other mechanisms of activation.

**Note.** While our manuscript was under review a phylogenetic analysis of the FH2 domain by H. N Higgs and K. J. Peterson has been published. These authors used a larger set of FH2 domains that includes only three formins from *Dictyostelium*. The topology of the phylogenetic tree described in that article and that of our tree are essentially coincident, and all seven metazoan groups identified by their authors can be found in our tree, with the novel subfamily INV comprising our HsKIAA1727, DmAE003560 and CeAF106580 sequences. Higgs and Peterson, however, do not recognize the GBD/FH3 region as a domain present in a larger number of formin subfamilies.

## Methods

### Sequence analysis

The amino acid or DNA sequences of *Dictyostelium *formins were used as query for BLAST searches [42] of the *Dictyostelium *genome project databases at The Welcome Trust Sanger Institute, Baylor College of Medicine, The University of Cologne and the Department of Genome Analysis of the Institute of Molecular Biotechnology in Jena. Nearly all of this data was generated at the aforementioned institutes with a small part of it produced at the Institute Pasteur. After assembly of the genome further analyses were performed through the Dictybase server [43]. BLAST searches against EST sequences were performed at NCBI [44]. Accession numbers for *Dictyostelium *formins can be found in Table 1.

Accession numbers of the sequences retrieved for phylogenetic analyses are as follows. *S. cerevisiae *Bni1p, P41832; Bnr1p, P40450; *S. pombe *Fus1, L37838; Cdc12, 786133; For3, AL035247. *D. melanogaster *Cappuccino, U34258; Diaphanous, U11288; FHOD, AE003554; FMNL, BT003654; DAAM, AAF45601; a novel formin, AE003560. *C. elegans *FHOD, U88314; Cyk-1, U40187; FMNL, AC024798; novel formins, Z78013, AF106580 and Z22174. *H. sapiens *Formin 1, AK127078; Formin 2, XM_351329; FHOD1, AF113615; FHOD3/FHOS2, KIAA1695; DRF1, AF05187; DRF2, Y15909; DRF3, BC034952; Delphilin, XM_353725; FMNL1, AF432213; FMNL2/FHOD2, KIAA1902; WBP3/FMNL3, NM_175736; DAAM1, NM_014992; DAAM2, AL833083; a novel formin KIAA1727. Sequences of plant formins were obtained from Cvrčková et al. [16]. *Dictyostelium *RasGEF-L and RasGEF-V can be accessed at Dictybase [43] under DDB0217789 and DDB0216586, respectively.

Protein sequences were aligned using the ClustalX [45] program with a BLOSUM62 matrix and default settings, followed by manual edition with the Bioedit program [46]. Phylogenetic trees were constructed using the neighbor-joining algorithms of the ClustalX program with correction for multiple substitutions; positions with gaps were not excluded. Construction of trees was done with TreeView [47]. Bootstrap analysis (1000 bootstraps) was applied to provide confidence levels for the tree topology. The domain analysis was done using the SMART tool [48] and InterProScan [49]. FH1 domains were identified by visual inspection. GBD/FH3 and DAD domains were identified in part by inspection of multiple alignments.

### Cell culture

*D. discoideum *AX2 strain was grown at 21°C in shaking suspension in axenic HL5 medium [50]. AX2 cells were also cultured for sexual gametes in Bonner's salt solution (BSS) as described [33]. In brief, cells were cultured for 15 hours in a dense suspension of *K. aerogenes *in BSS either in the darkness or in the light. Cells cultured for 15 hours in the dark become fusion-competent cells. However, cells cultured for 15 hours in the light condition exhibit reduced fusion competency, and are designated light submerged cells. Cells on SM agar plates [50] are fusion incompetent cells.

### Isolation of total RNA and quantitative real-time PCR

Total RNA was purified from both asexually and sexually developing cells with the TRIZOL reagent (GIBCO BRL, USA). Asexually developing cells on phosphate agar plates [50] were collected every 4 hours. Total RNA was treated with RNase-free DNase to remove contaminating genomic DNA, and then used to synthesize the first strand cDNA using SuperscriptII (Invitrogen, USA). For each time point cDNA was synthesized using two independently isolated mRNA samples. Specific primer sets for each formin gene were designed. To equalize the concentrations of template cDNAs, amplification was conducted using the control primer set for the Ig7 gene, which is expressed constitutively. Quantitative real-time PCR was performed with an ABI 7900HT Sequence Detection System according to the manufacturer's instructions. The amplifications were carried out using Ex Taq R-PCR Version (Takara Bio, JAPAN) and SYBR Green I. Each sample had 2 replicates containing 1-, 4-, or 16-fold diluted cDNA.

### Miscellaneous methods

For RT-PCR, first strand cDNA synthesis was performed with M-MLV reverse transcriptase (Promega Corporation, Madison, WI) on poly A+ mRNA purified with the Oligotex system (Qiagen GmbH, Hilden, Germany) from total RNA. PCR fragments were cloned into the pGEM-T Easy vector system (Promega Corporation, Madison, WI) and sequenced. DNA sequencing was done at the service laboratory of the Center for Molecular Medicine, Cologne, using an automated sequencer (ABI 377 PRISM, Perkin Elmer, Norwalk, CO).

## Authors' contributions

FR conceived the study, performed the assembly of genomic sequences and the sequence alignments and drafted the manuscript. TM and HU carried out the gene expression studies. AKM performed RT-PCR and cloning. CK and TQPU participated in the design of the study and the assembly of genomic sequences. All authors read and approved the final manuscript.
